# Evaluating Gene Expression Dynamics Using Pairwise RNA FISH Data

**DOI:** 10.1371/journal.pcbi.1000979

**Published:** 2010-11-04

**Authors:** Matthieu Wyart, David Botstein, Ned S. Wingreen

**Affiliations:** 1 Lewis-Sigler Institute for Integrative Genomics, Princeton University, Princeton, New Jersey, United States of America; 2 Center for Soft Matter Research, New York University, New York, New York, United States of America; 3 Department of Molecular Biology, Princeton University, Princeton, New Jersey, United States of America; Duke University, United States of America

## Abstract

Recently, a novel approach has been developed to study gene expression in single cells with high time resolution using RNA Fluorescent In Situ Hybridization (FISH). The technique allows individual mRNAs to be counted with high accuracy in wild-type cells, but requires cells to be fixed; thus, each cell provides only a “snapshot” of gene expression. Here we show how and when RNA FISH data on pairs of genes can be used to reconstruct real-time dynamics from a collection of such snapshots. Using maximum-likelihood parameter estimation on synthetically generated, noisy FISH data, we show that dynamical programs of gene expression, such as cycles (*e.g.*, the cell cycle) or switches between discrete states, can be accurately reconstructed. In the limit that mRNAs are produced in short-lived bursts, binary thresholding of the FISH data provides a robust way of reconstructing dynamics. In this regime, prior knowledge of the type of dynamics – cycle *versus* switch – is generally required and additional constraints, *e.g.*, from triplet FISH measurements, may also be needed to fully constrain all parameters. As a demonstration, we apply the thresholding method to RNA FISH data obtained from single, unsynchronized cells of *Saccharomyces cerevisiae*. Our results support the existence of metabolic cycles and provide an estimate of global gene-expression noise. The approach to FISH data presented here can be applied in general to reconstruct dynamics from snapshots of pairs of correlated quantities including, for example, protein concentrations obtained from immunofluorescence assays.

## Author Summary

Programs of gene expression lie at the heart of how cells regulate their internal processes. Some dynamical gene-expression programs, such as the cell cycle, are well known and studied, others, such as metabolic cycles, have only recently been recognized, and many other dynamical programs including switches are likely to be discovered. Traditional bulk studies typically fail to resolve such cycles or switches, because individual cells are out-of-phase with each other. On the other hand, standard techniques for studying single cells are limited in time resolution and scope. RNA Fluorescent In Situ Hybridization (FISH) is a single-cell technique that offers both high time-resolution and precise quantification of mRNA molecules, but requires fixed cells. We have explored how, when, and with what prior information FISH snapshots of pairs of genes can be used to accurately reconstruct gene-expression dynamics. The technique can be readily implemented, and is broadly applicable from bacteria to mammals. We lay out a principled and practical approach to extracting biological information from RNA FISH data to reveal new information about the dynamics of living organisms.

## Introduction

Cells are well known to respond to external conditions by altering their gene expression. In recent years, many examples of altered gene expression programs have been revealed by population level studies, including microarray studies of yeast, mammalian, and bacterial cells. But many cells are also known to alter gene expression is ways that are heterogeneous across a cell population. Examples include the acquisition of competence for DNA uptake [1], [2] and spore formation [3] in *Bacillus subtilis*, induction of the *lac* operon in *Escherichia coli* depending on “memory” of previous exposure to lactose and the presence of lactose permease [4], [5], and the response of *Saccharomyces cerevisiae* (budding yeast) temperature-sensitive mutants to a shift to non-permissive temperature depending on the position of cells in their division cycle [6], [7]. Heterogeneous changes in gene expression in response to homogeneous external cues may be purely stochastic as in the switch to competence in *B. subtilis* [1], [2], [8], or may depend on pre-existing non-genetic differences such as the phase of the cell cycle in budding yeast [6], [7].

Since population level studies are not well suited to reveal heterogenous behavior, how can heterogeneous changes in gene expression be studied and quantified? Fluorescent reporter proteins have been used successfully to report on expression of a small number of genes either via FACS analysis or fluorescence microscopy. However, the use of fluorescent reporters is generally limited to highly expressed genes, with time resolution severely limited by fluorescent protein maturation and the low turnover rates of the fluorescent marker. Moreover, construction of fluorescent reporters can be laborious and impractical for studies of large-scale transcriptional responses.

A promising approach that has recently been used to study gene expression on a cell-by-cell basis is Fluorescence In Situ Hybridization (FISH) [9]–[11]. In FISH, fixed cells are exposed to fluorescently labeled probes of specific mRNA transcripts, so that the number of these mRNAs can be counted in each cell by the number of bright spots. Advantages of FISH include: (1) absolute quantification since the actual number of mRNAs can be counted, (2) time resolution since there is no delay for reporter maturation, (3) ability to directly study wild-type cells, and (4) the ability to probe simultaneously for multiple mRNAs, *e.g.* by employing probes with different fluorescent spectra [10], [12]. A significant disadvantage of FISH is the requirement to fix cells. This disadvantage presents a particular challenge when it is the dynamics of gene expression that is of central interest. For example, each individual drawn from an asynchronous yeast population represents a particular moment in the cell division cycle. In essence, the problem we wish to address is how to reconstruct the dynamics of gene expression from what amount to “snapshots”, where each individual cell represents a different point in time.

**Figure 1:**
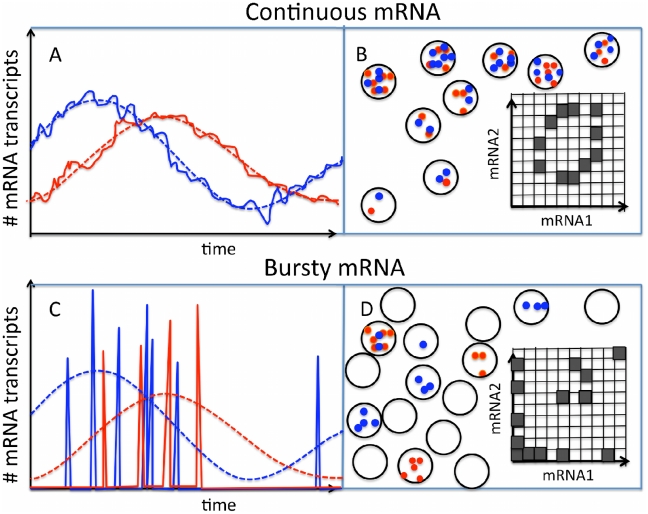
Illustration of periodic mRNA transcription and pairwise FISH measurements in the continuous regime (upper panel) and the bursty regime (lower panel). The dashed curves indicate mean transcript numbers. The actual number of mRNA transcripts will fluctuate from cell to cell and from cycle to cycle. (A) Sketch of the number of mRNA transcripts versus time for two genes in the continuous regime, where fluctuations about the mean are small. (B) Sketch of FISH observations corresponding to (A): a large number of mRNA transcripts from both genes will typically be found in each cell. Inset: schematic of corresponding distribution of pairwise FISH data. (C) Sketch of the bursty regime where typically only the most recent transcriptional burst contributes to the total mRNA number, implying large fluctuations about the mean. (D) Sketch of FISH observations corresponding to (C): many cells display either no mRNA or bursts of a single mRNA type, and coincident bursts of both mRNA types are rare. Inset: schematic of corresponding distribution of pairwise FISH data.

Here, we present an approach to extracting information about the dynamics of gene expression from FISH data by considering correlations of expression between pairs of genes (*cf.* Fig. 1). The approach applies even if the dynamics of interest occurs heterogeneously in a population. One class of dynamics we consider are cyclic oscillations of gene expression. Common examples are the cell cycle, circadian oscillations, and metabolic oscillations [13]. Cyclic oscillations of gene expression, such as the cell cycle, have been studied at the population level by synchronizing cells, but for many organisms synchronization is difficult without strongly perturbing the cells. A non-perturbative approach to studying oscillatory gene expression is likely to be of value in these cases. To study metabolic oscillations, cells of the yeast *Saccharomyces cerevisiae* have been synchronized in chemostats [13], but those cells demonstrably continue to influence each other via levels of dissolved oxygen and other chemical species. To ascertain if *Saccharomyces* undergoes metabolic oscillations outside the chemostat, Silverman *et al.* [14] recently obtained an extensive FISH data set, and argued for the existence of metabolic oscillations based on correlations in gene expression. Using the same data set, we apply our approach to reconstructing oscillatory dynamics, and confirm the existence of metabolic cycles in unsynchronized yeast populations [14]. Our approach can also be applied to transient oscillations in response to external stimulation, such as in the bacterial SOS response to DNA damage [15] or in the analogous eukaryotic p53-Mdm2 system [16]. Another class of dynamics we consider are stochastic switches among different states of gene expression. Examples include persister cells in *Escherichia coli* [17], competence [1], [2], [8] and swimming/chaining in *Bacillus subtilis* [8], the stringent response in mycobacteria [18], and galactose utilization in *Saccharomyces cerevisiae* [19].

Specifically, we show how Maximum Likelihood Estimation (MLE) [20] can be applied to FISH data obtained for multiple pairs of genes to reconstruct the underlying dynamics of gene expression. MLE consists of finding the set of parameters within a particular family of models for which the observed data is most “likely”. MLE has been applied successfully to biological data analysis in many contexts, from reconstruction of evolutionary trees [21], [22] to estimation of genetic parameters [23] to understanding the evolution of gene structure [24]. We show using synthetic FISH data that MLE can accurately reconstruct dynamics, even in the presence of substantial noise, provided the number of genes and the number of FISH observations per gene pair are sufficient. Reconstructing gene-expression dynamics is most challenging in the “bursty” regime where mRNAs are often present at very low levels or not at all in the cell, except when transcriptional bursts occur. For this regime, we present a robust approach based on thresholding the FISH data into binary form, followed by MLE analysis. In this case, we show that Principal Component Analysis (PCA) of the covariance matrix performs nearly as well as MLE. We suggest that the two-step approach of thresholding followed by MLE or PCA is likely to prove the best practical approach to reconstructing gene-expression dynamics for most real FISH data sets, and we demonstrate this approach using the data set of Silverman *et al.* [14].

Importantly, the method we present here for inferring intracellular dynamics from data in the form of “snapshots” is quite general, relying only on measurements of pairs of quantities in single cells, with no requirement for exact counts. The method can therefore be applied with little modification in other contexts such as quantitative immunofluorescence or single-cell sequencing studies.

## Results

We presume that production of mRNA transcripts is a stochastic process. Transcription factors bind to DNA at random times, with a probability that depends on other signals, and which can therefore also vary with time. Binding of one or more transcriptional activators, or unbinding of repressors, typically leads to production of a “burst” of mRNA transcripts. One can distinguish three regimes, two of which are illustrated in Fig. 1. In the first regime, many bursts typically contribute to the total concentration of a particular mRNA species at any moment. The distribution of mRNA is therefore approximately Gaussian with a mean and variance that can vary with time, *e.g.* over the cell cycle. We refer to this case as the continuous regime. The second regime is the opposite limit where mRNA production is highly intermittent [10] – typically there are very few mRNAs of a particular species, and when there are more than a few, they all stem from the same burst. We refer to this case as the bursty regime. The third regime is the intermediate case, where a few bursts typically contribute to the number of mRNA present at any moment. In what follows we focus on the two first regimes. Optimal treatment of the intermediate regime requires a more detailed and/or empirical noise model, but the thresholding method we develop for the bursty regime can also be usefully applied in the intermediate case, as demonstrated by our analysis of FISH data for metabolic cycles in yeast [14].

For each regime of mRNA expression, our approach consists of defining a class of possible dynamics, and choosing the one for which the observed data is most likely. Specifically, for a given set of model parameters, we calculate the probability of the observed data, and then ask for the particular set of parameters that maximizes this probability. Since the probabilities don't sum to one over all models (*i.e.* sets of parameters), they are called “likelihoods” and hence this approach to parameter inference is called Maximum Likelihood Estimation (MLE). Below, we demonstrate the practicality of the MLE approach using synthetically generated FISH data in both the continuous and bursty mRNA regimes.

In practice the parameter optimization in MLE can be a challenge, and algorithms used to search parameter space for the maximum likelihood can get stuck in local maxima. However, the general formulation of the maximum likelihood approach is conceptually distinct from the detailed choice of algorithms used to optimize parameters, and so we have chosen to present only fully optimized results in the main text. In Methods, we present a practical method for searching parameter space that typically quickly finds the model parameters that maximize the likelihood of the data.

It is important to recognize one absolute limitation of using FISH data to reconstruct the dynamics of gene expression. Because cells must be fixed before mRNAs are measured, only “snapshots” of individual dynamical trajectories are available. As a consequence, it is impossible from FISH data alone to determine the overall time scale of the dynamics of gene expression. Thus, while it is possible to infer from correlated FISH data that cells undergo cycles of gene expression, and even practical, as we will show, to accurately reconstruct such cycles, it is not possible, even in principle, to determine the period of these oscillations. Similarly, it is not possible, even in principle, to determine which direction around the cycle of gene expression corresponds to the forward arrow of time. In many cases, we anticipate that other methods, *e.g.* fluorescent reporters or population-level assays, can be used to provide this missing information. In some cases, the insensitivity of FISH data to cycle period may actually prove advantageous. In bulk studies of synchronized cell populations, different cycle periods of individual cells lead to loss of synchrony and therefore loss of signal. In contrast, for single-cell FISH studies, differences in cycle period among cells will not affect mRNA correlations. Hence, variations of period will not affect the ability to reconstruct cycle dynamics from mRNA snapshots. However, cell-to-cell variations of the shape of the cycle constitute noise even for FISH, which can at best allow reconstruction of the mean cycle waveform.

**Figure 2:**
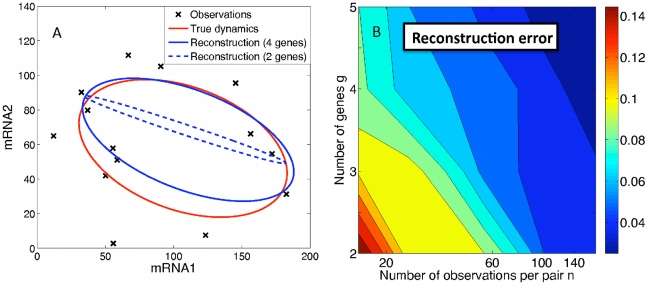
Maximum likelihood estimation (MLE) applied to synthetic FISH data in the continuous regime. (A) Comparison of true mRNA dynamics (red curve) with MLE reconstructions based on 4 genes (solid blue curve) and 2 genes (dashed blue curve), for 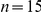 observations per gene pair. The data points are the actual synthetic data used for genes 1 and 2 in both cases. The maximum noise amplitude used to generate the data is 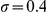. (B) The reconstruction error 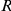, averaged over 20 realizations of parameters with 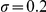, steadily decreases as the number of genes 
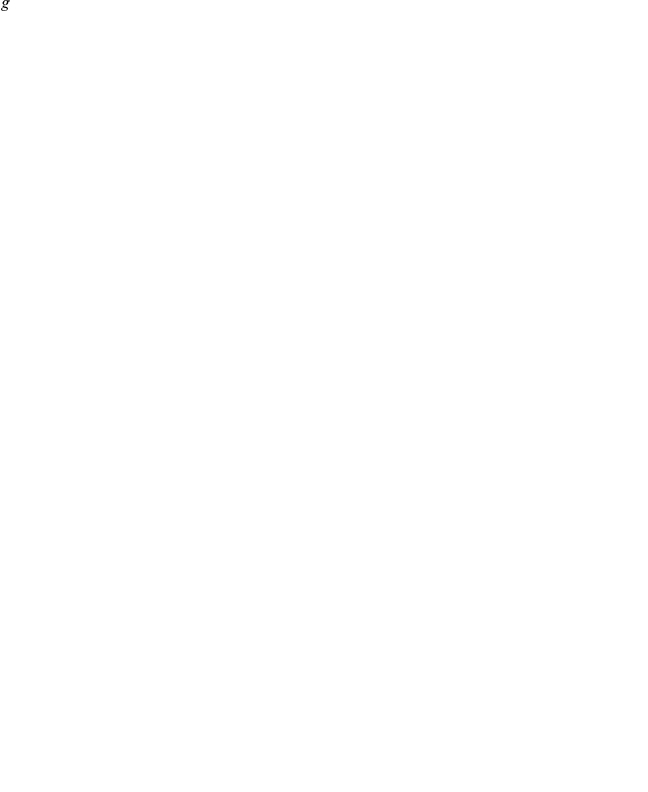
 and the number of FISH observations 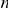 per gene pair are increased.

At a qualitative level, the regime of continuous mRNA production allows for relatively straightforward reconstruction of cyclic gene-expression dynamics. In the absence of noise, FISH data for even a single pair of genes is sufficient. One can simply plot the ordered pairs of FISH data as in Fig. 2A, and infer the dynamics from the smooth trajectory that joins the data points. (The fundamental limitations of FISH are already clear in this case – from the FISH data points alone one cannot in principle infer the period of the trajectory nor its direction.) Noise complicates the reconstruction somewhat, and requires a computational means of inferring the trajectory that best fits the data. Our solution presented below is to find the trajectory most likely to account for the data, within a family of harmonic functions, 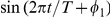, 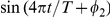, etc. The ability to accurately reconstruct trajectories in the presence of noise is greatly improved by FISH data for multiple pairs of cycling genes. Geometrically, the true trajectory is a path in the space of all the cycling genes. Each set of pairwise FISH data represents a projection of this trajectory onto a plane as in Fig. 2A. The more such projections are available, *i.e.* the more sets of pairwise FISH data, the more accurate the reconstruction of the true trajectory will be. This approach can be readily extended to the case of a stochastic switch between distinct gene-expression states, with the same improvements expected from multiple FISH pairs.

Reconstruction of gene-expression dynamics in the regime of bursty mRNA production is more challenging. In this case, the data consists of the presence or absence of bursts of mRNAs, with rare coincidences of bursts for two genes (*cf.* Fig. 1D). All of the information in the data is therefore captured by a single number for each pair of genes, namely the covariance of their mRNA bursts. However, as we show below, the matrix of these covariances for multiple gene pairs in principle contains enough information to reconstruct the underlying parameters of cyclic trajectories or stochastic switches (albeit in some case with degeneracies that require additional constraints to resolve). Since coincident bursts of mRNAs are likely to be rare, one expects the covariance matrix derived from the data to be noisy. Nevertheless, with a sufficient number of sets of pairwise FISH data, we find that accurate reconstruction of the underlying gene-expression dynamics is feasible.

### Continuous production of mRNA

We first consider the continuous regime where many bursts typically contribute to the instantaneous mRNA number. To demonstrate the MLE algorithm, we reconstruct the dynamics of gene expression using synthetic FISH data for which the underlying dynamics is known. We focus on analyzing cyclic dynamics, *e.g.* the cell cycle or a metabolic cycle; the results can be readily extended to stochastic switches, which are introduced in a later section. We denote the mean expression level of mRNA for gene 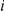 by 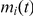, which is taken to be periodic with the same period for genes 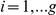. For concreteness, we denote the period as 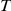, although 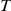 cannot be inferred from FISH data alone. FISH observations 
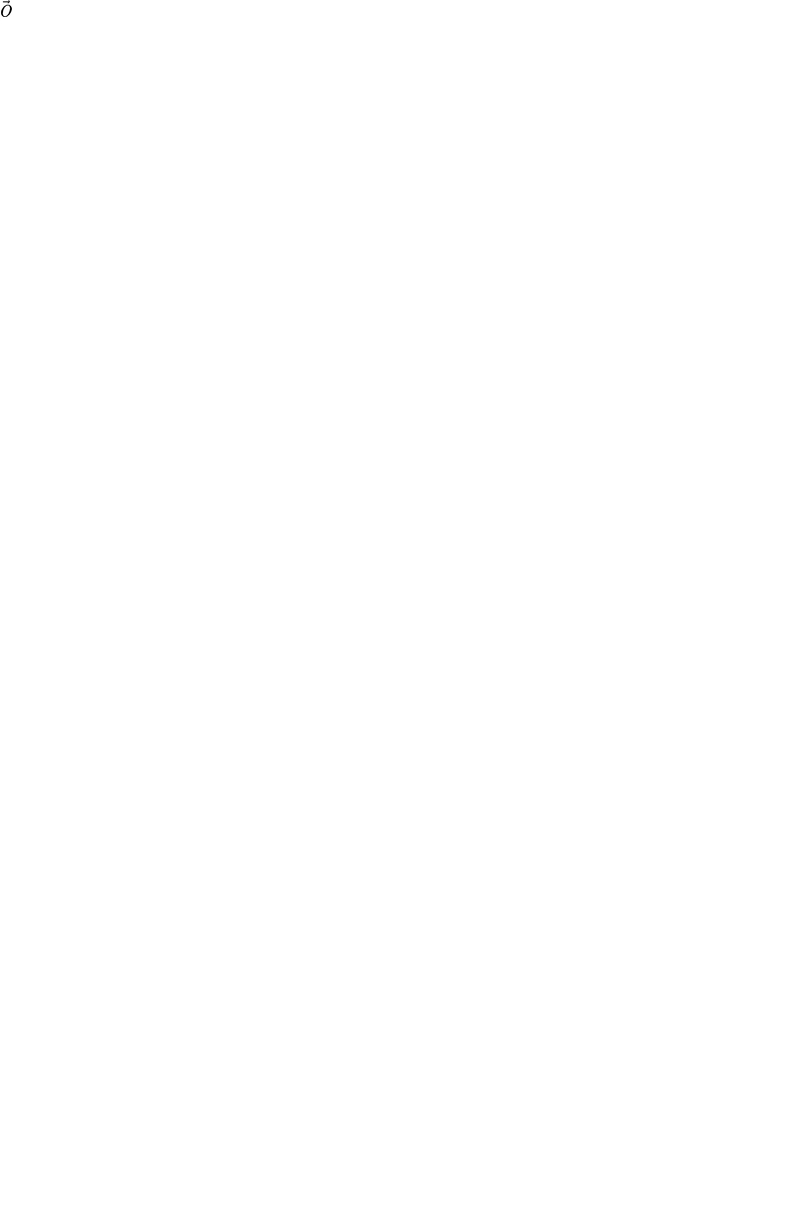
 are generated for pairs of genes at randomly chosen times: 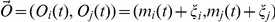, where the 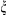 s reflect fluctuations in mRNA number around the mean, as well as noise in the measurement. 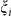 is assumed to be a Gaussian random variable of mean zero and standard deviation 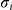. We assume that 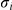 is not a function of the mean expression 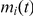, but it is straightforward to extend the method to the more general case. (A natural extension of the model is to consider 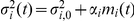 where 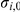 characterizes the measurement noise and 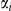 is the characteristic size of the independent events of mRNA production leading to the total mRNA number.) We aim at maximizing the likelihood of the observations within a family of harmonic functions of period 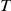. Bayes Theorem for the probability 
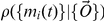
 of a particular model (*i.e.* set of parameters) given the data states:
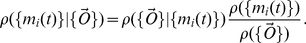
(1)We neglect the term, 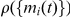, corresponding to prior knowledge of the parameters, as there is no obvious choice for what such prior knowledge should be; moreover, with sufficient data, including such priors generally has little effect on the results of optimization. The probability of the data 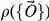 is a constant normalization factor, and so does not affect the relative likelihood of models. Therefore the probability of the model given the observations is proportional to the probability of the observations given the model 
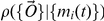
.

For each FISH observation, one therefore has:
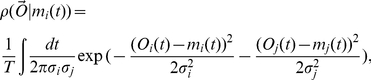
(2)and for the combined likelihood 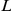 over all observations,
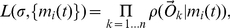
(3)where the product runs over all 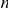 FISH observations. In what follows we maximize 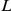 assuming harmonic oscillations of mRNA levels,
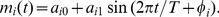
(4)The method can be systematically extended to periodic trajectories that are not simple sine waves by including higher harmonics. It is also straightforward to extend the method to more detailed noise models. For example, non-Gaussian noise can be incorporated by appropriately modifying the Gaussian integrand in Eq. (2). Similarly, global transcriptional noise [25] can be modeled in Eq. (2 via a single additional random variable multiplying both 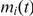 and 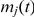. Later, we consider both higher harmonics and global noise in detail for the more physiologically relevant case of bursty mRNA production.

### Synthetic data

We generate synthetic FISH data by first choosing the parameters in Eq. (4) for the oscillating mRNA levels 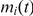, and then generating FISH observations based on these parameters. Specifically, we choose random variables 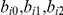 uniformly on 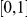, for genes 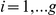. We then define the model parameters in Eq. (4) as 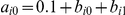, 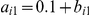 and 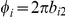. This construction ensures the positivity of the mRNA levels 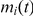, and also ensures that the genes considered oscillate in time with significant amplitudes. The noise amplitudes 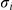 are random variables, distributed continuously, 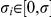. The synthetic FISH data are generated by choosing for each gene pair 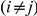, 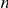 random times 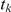 and 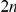 random noise values 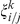, and constructing 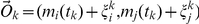. In this way, the synthetic data correspond to a set of independent, pairwise FISH observations. An example is shown in Fig. 2 for 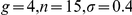. The red ellipse indicates the true mean-mRNA-level trajectory 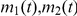, and the crosses are the randomly generated FISH data points. The blue ellipses correspond to reconstructions of the mean trajectory via maximization of the likelihood in Eq. (3).

### Reconstruction of mRNA dynamics

To test the accuracy of reconstruction of mRNA dynamics using our MLE approach, we generated a large number of sets of parameter, and for each parameter set generated synthetic FISH data and then applied MLE to reconstruct the true dynamics. Specifically, for each parameter set defining a trajectory of mean mRNA levels 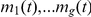, we maximized the likelihood 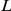 with respect to 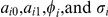. To ensure that we always found the global maximum of the likelihood, the initial guess for the parameters was taken to be the true parameters describing the mean dynamics. (In Methods, we present a simple algorithm that almost always finds the global likelihood maximum without prior knowledge of the true parameters. However, in Fig. 2, we chose to present the true MLE optimum as the fundamental limit of reconstruction accuracy, not limited by a particular algorithm.)

As shown in Fig. 2, with synthetic FISH data for only two genes (dashed blue ellipse) the reconstruction is rather poor, in this case mistakenly assigning too large a noise 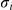 to each gene and missing the phase shift. However, the addition of pairwise information from two more genes to make 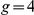 (for a total of 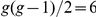 gene pairs) is enough to correct these errors and provide a very accurate reconstruction. Since each pairwise data set is independent, the total amount of data grows as 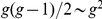.

To quantify the accuracy of the MLE algorithm, we computed the reconstruction error 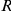, which characterizes how much the reconstructed dynamics varies from the true mRNA dynamics,
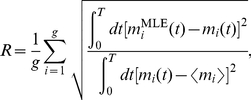
(5)where 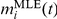 is the MLE reconstructed trajectory for mRNA 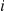, and 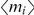 is the (true) average number of mRNA 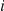 over the period 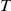. Results for the reconstruction error are shown in Fig. 2B for 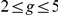, 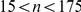, and 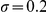. Each point is averaged over 20 randomly generated parameter sets. As expected, the results improve with the number of genes 
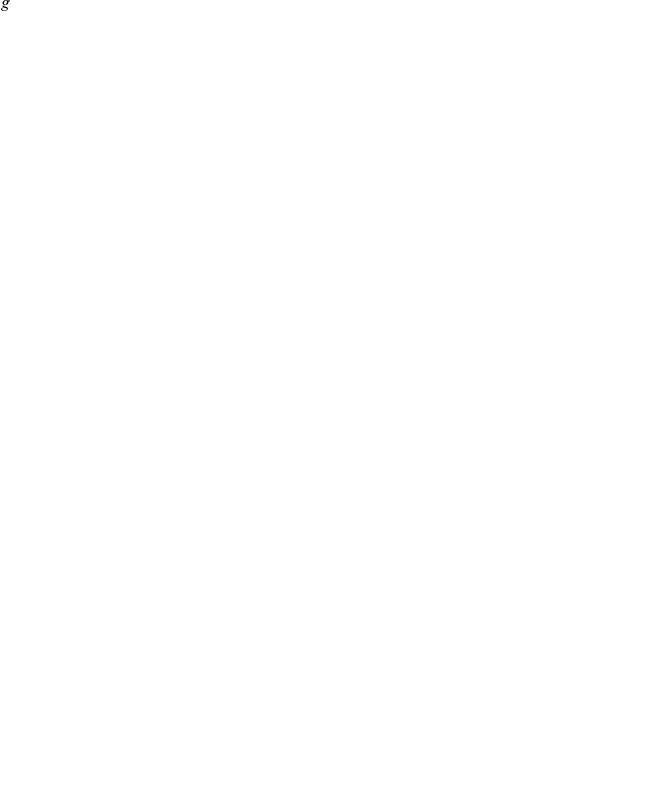
 and the number of FISH observations 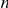 per gene pair, but at this noise level the results are already good 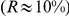 for 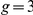 and 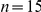.

### Bursty production of mRNA

We now consider the bursty regime where a cell will typically either have few (or no) mRNAs of a particular type, or the mRNAs present will come from a single recent burst of transcription. In this limit, the information provided by FISH is essentially binary - either mRNAs for a particular gene are present at significant levels, indicating a recent burst, or they are not. Formally, if a significant number of mRNAs for gene 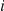 are present, then 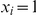, otherwise 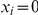. The optimal threshold 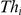 to set for the “presence” of mRNAs 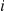 will depend on burst size and duration, measurement noise, and the total number of FISH observations – see Discussion. FISH data yields an estimate 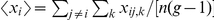 for the mean probability that mRNAs are present above the threshold value 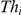 for each gene (in the expression for 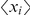, the variable 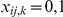 reports the absence or presence of mRNA 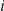 for observation 
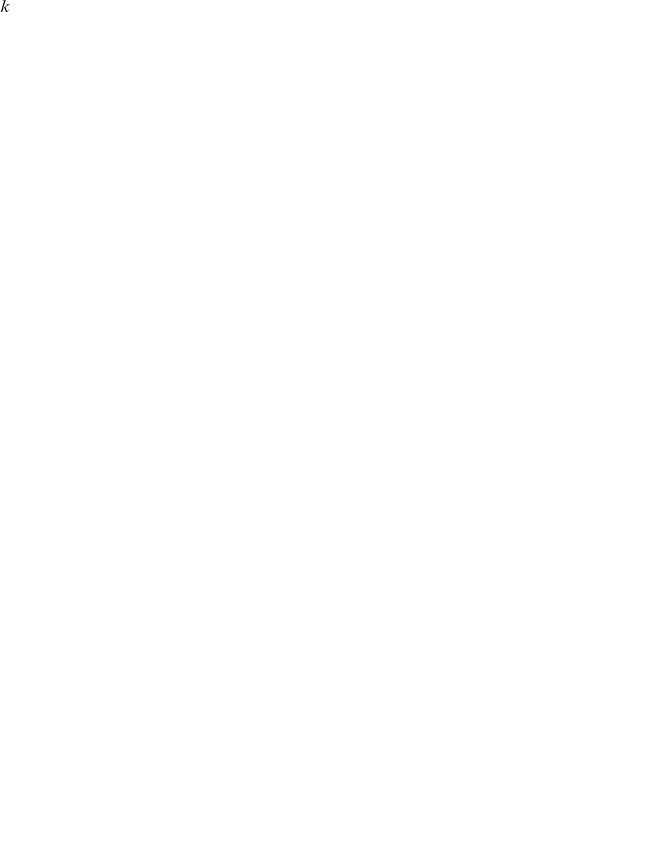
 of the pair 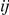, and the sum is made on the 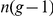 observations that probe for mRNA of gene 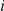.) The FISH data also yields an estimate for the covariance 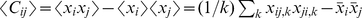 for each pair of genes. We aim to accurately reconstruct the mRNA dynamics from these quantities 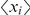 and 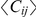, which capture all the information provided by the binarized FISH data in the bursty regime. However, even with perfect knowledge of mean expression and covariance, the reconstruction of mRNA dynamics has fundamental limitations in this regime. We illustrate by considering both cyclic dynamics and stochastic switches.

### Cyclic dynamics

We denote by 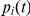 the probability that the number of mRNAs of type 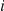 present at time 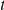 is larger than some threshold 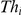, and we call such an event a burst in what follows. Assuming 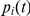 is any periodic function with period 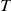, it can be expanded in harmonics:
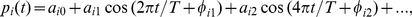
(6)with more harmonics generally required to capture more complex oscillation patterns. Note that if 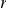 is twice the number of harmonics considered, the number of parameters is 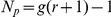, the 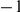 coming from the invariance with respect to the overall phase. For the following discussion it is sufficient to keep only the first two harmonics, shown explicitly in Eq. (6). In this case, denoting by 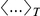 the average over a cycle, one finds:
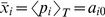
(7)

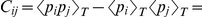


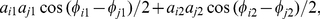
(8)where 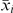 and 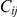 denote the true cycle-averaged mean and covariance, respectively. One immediately sees that the transformation 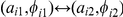 for all 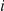 leaves both 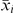 and 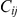 unchanged. Thus, in this bursty regime, pairwise FISH data alone cannot disentangle different harmonics without prior knowledge. However, any additional constraint, including even a single triplet FISH data set, can readily resolve the ambiguity between harmonics. (A triplet FISH observation, *i.e.* simultaneous measurement of three different mRNA types, leads to terms 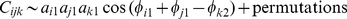, which do not have the problematic symmetry.)

For simplicity, let us now consider only the lowest harmonic, as in the previous section. We introduce the 
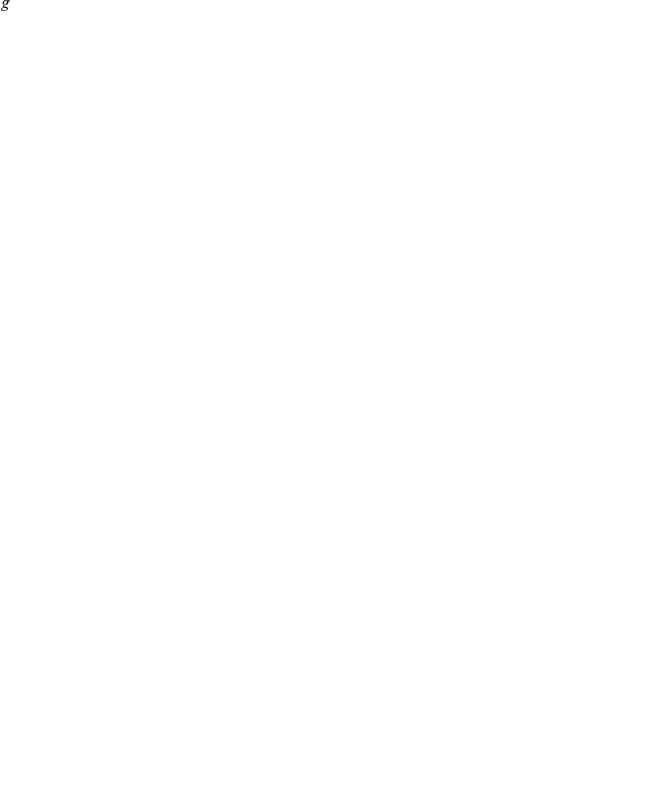
-dimensional vectors 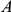 and 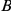, defined as 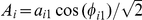 and 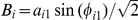. Each component can vary independently of the others, as there are 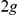 parameters, and 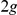 coordinates for the two vectors. Then by inspection the covariance matrix 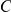 from Eq. (7) can be written:
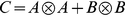
(9)which shows that 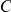 is in general of rank 2. If the second harmonics are included, 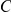 is of rank 4, etc. Note that all symmetric matrices of rank 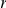 can be written in the form of Eq. (9), with 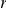 eigenvectors, implying that for a covariance matrix of even rank there is always an interpretation of the dynamics in terms of cyclic trajectories. Unfortunately, this interpretation is not unique except for the case of a single harmonic (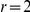), which can be seen as follows. The observed mean probabilities for mRNA bursts of each type leads to 
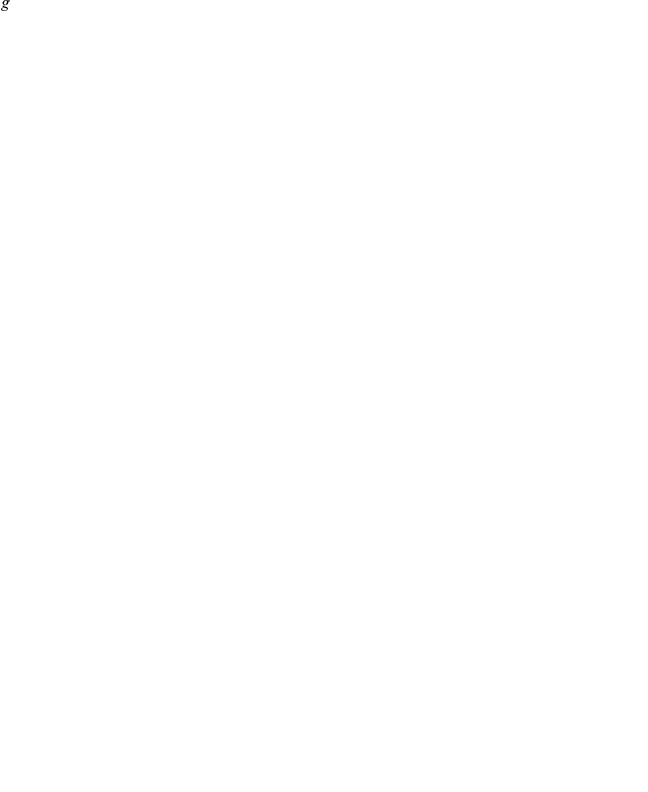
 constraints. The observed covariances lead to an additional 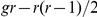 constraints. Being a symmetric matrix of rank 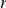, the covariance matrix can be defined by this many coefficients, *i.e.* the number necessary to describe the 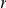 eigenvectors, enforcing orthogonality among them. The expression for the number of covariance constraints is true for sufficient large 
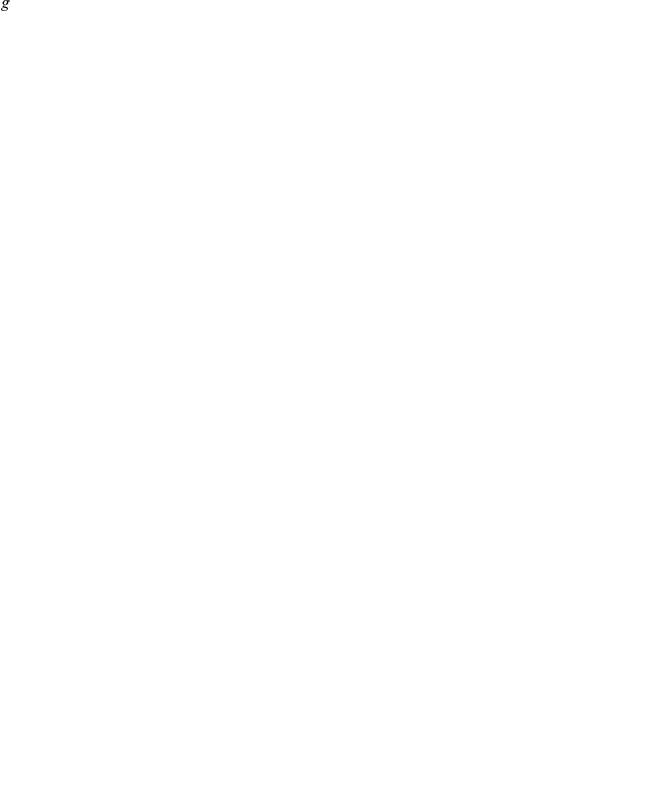
, but in general is 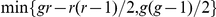.) The total number of constraints provided by FISH is therefore 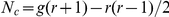. Thus the number of unconstrained parameters is 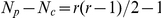, which is zero for 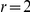, but is already 5 for 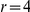. Hence, for two harmonics 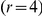 at least 5 triplet FISH data sets or other constraints are required to be able to infer all the parameters.

An important consideration in analyzing FISH data is that overall transcription rates may vary from cell to cell. Indeed, measurements of gene-expression noise in single yeast cells at the protein level reveal 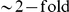 global fluctuations [25]. How can the dynamics of bursty gene expression be reconstructed against the background of these global correlations? We consider the case of a simple harmonic cycle. The probability 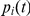 that the number of mRNAs of type 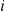 present at time 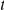 is larger than some threshold 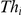 now reads:
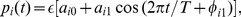
(10)where 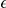, representing the fluctuating global level of transcription, is a random variable of mean unity and standard deviation 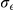. One then obtains for the true cycle-averaged mean 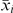 and covariance 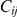:
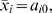
(11)

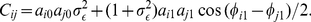
(12)Introducing the definitions 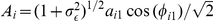, 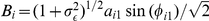, and 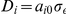, the covariance matrix 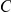 from Eq. (12) can now be written:
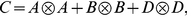
(13)which shows that 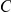 is now of rank 3. If the second harmonics are included, 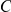 is of rank 5, etc. The maximum likelihood reconstruction for the model of Eq. (10) provides an estimate of the level of global transcriptional noise, as we show below for our reconstruction of metabolic cycles in yeast.

### Switching dynamics

**Figure 3:**
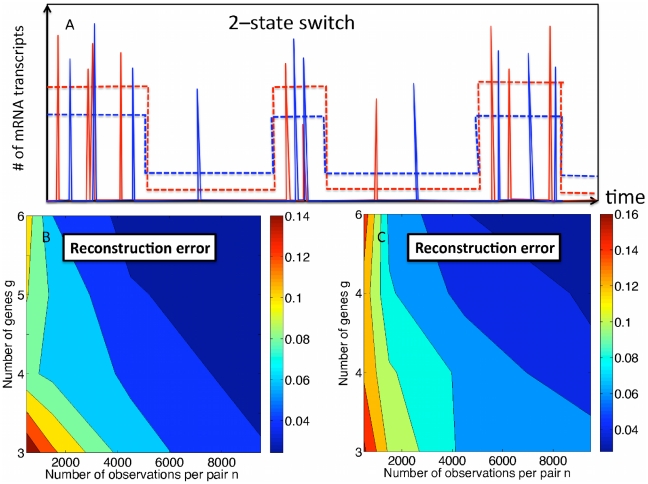
A stochastic switch in the regime of bursty mRNA production. (A) Illustration of the number of mRNA transcripts versus time, in the bursty limit, for two genes subject to regulation by a stochastic 2-state switch (solid lines); dashed lines indicate true burst probabilities 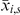 in each state. Reconstruction error 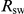 averaged over 20 realizations of parameters as number of genes 
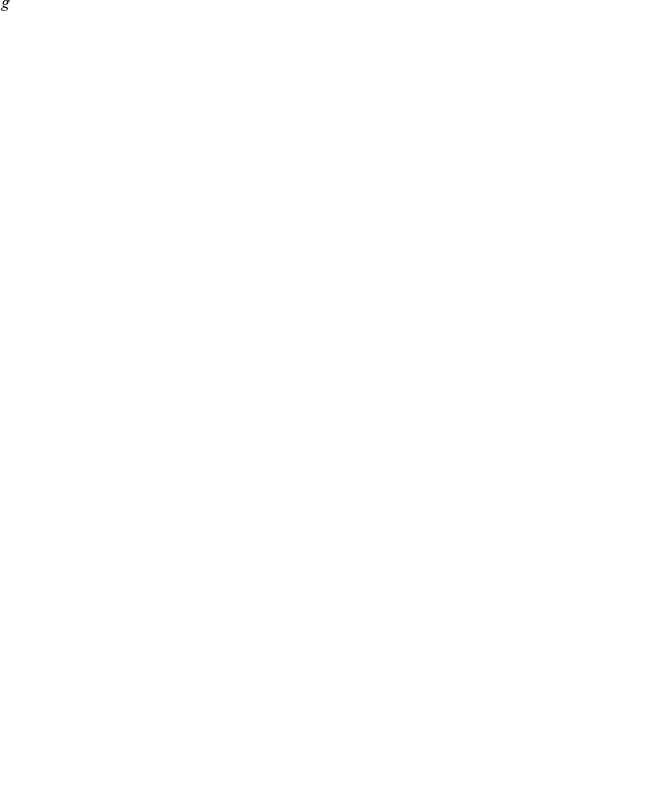
 and number of FISH observations 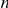 per gene pair are varied, for (B) maximum likelihood estimation, and (C) principal component analysis (PCA). The probability of the states was chosen as 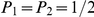, and the parameters 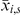 were chosen randomly on 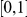. To ensure that all genes considered are informative about the state of the switch, the constraint 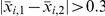 was enforced.

We now consider a model where the expression pattern can switch stochastically among 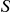 distinct states, as illustrated in Fig. 3. We assume all genes of interest switch their expression synchronously, consistent with control by a single transcription factor, and without delays, consistent with state lifetimes long compared to mRNA lifetimes. On average, each state 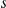 occurs with probability 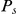. In state 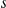, the true probability that a burst of mRNAs of type 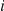 is present is denoted 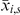. For such models, the number of parameters is therefore 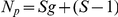, taking into account that 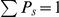. One finds, following simple arithmetic:
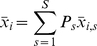
(14)

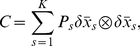
(15)where 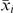 is the state-averaged burst probability and 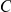 is the covariance matrix. 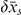 is a 
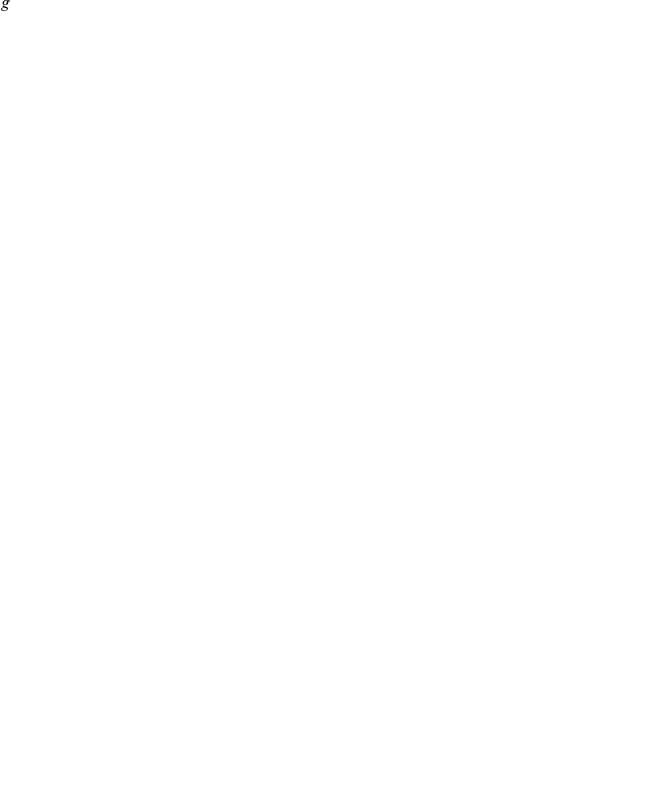
-dimensional vector of components 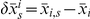.

It is straightforward to see that 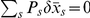, which implies that the different vectors 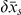 are not independent. Together with Eq. (15), this dependence implies that the covariance matrix 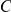 is in general of rank 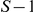. Thus, pairwise FISH data alone cannot distinguish a 3-state model from simple harmonic dynamics (or a 5-state model from a cycle including second harmonics, and so on). Moreover, even if one assumes that the dynamics is a switch, the parameters cannot be resolved uniquely: the number of constraints set by the measured means and covariances is 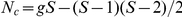, so that the number of unconstrained parameters is 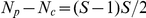, which is 1 for a 2-state switch, and 3 for a 3-state switch. The corresponding number of triplet FISH data sets or other constraints are therefore required for parameter inference; however, if this additional data is available, switching parameters can be inferred even in the presence of global noise, as discussed above for the case of a simple cycle.

### Maximum likelihood estimation

For either cyclic or switching dynamics, maximum likelihood parameter estimation in the regime of bursty mRNA production requires the following steps, (1) estimating the mean burst probability and covariance from the FISH data, (2) determining the uncertainty of these estimates, and (3) obtaining the parameters for which the observed data is most likely. Taking an average over FISH data provides an estimate 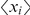 of the cycle- or state-averaged probability for a burst of mRNAs of type 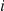 to be present. Specifically, 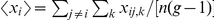, where 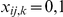 reports the absence or presence of mRNA 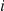 for observation 
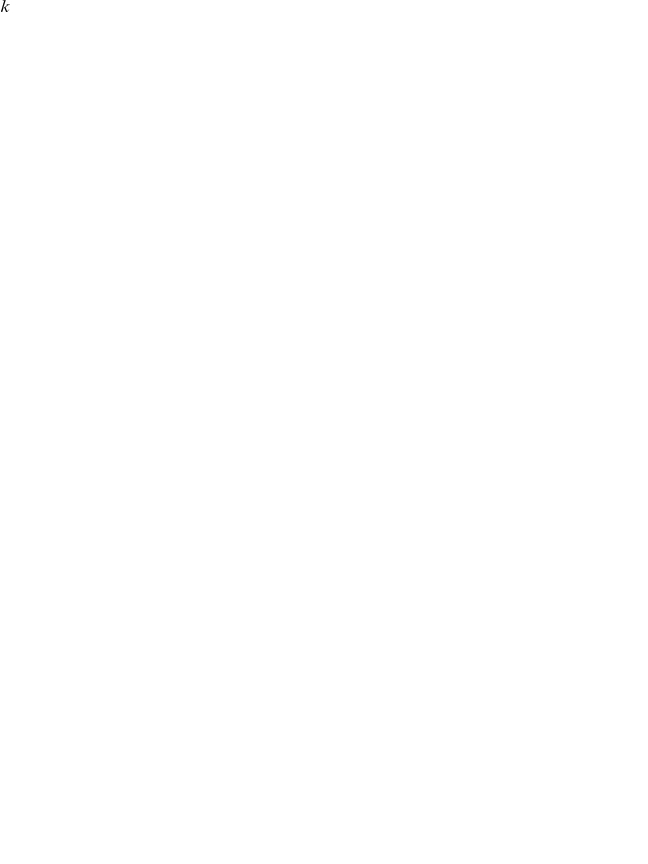
 of the pair 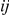, and where the sum is made on the 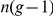 observations that probe for mRNA of gene 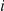. Similarly, FISH data provide an estimate of the covariance, namely 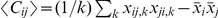. For a finite number of data points, these estimates will be noisy, *i.e.* 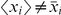 and 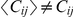, where the right hand sides are the exact values. Since coincident bursts of mRNAs of type 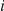 and 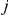 will be rare, the covariance estimate 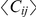 from finite FISH data may deviate significantly from the true covariance 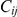, and one must allow for this uncertainty in the maximum likelihood calculation. In contrast, one may safely neglect the uncertainty in the FISH estimate of the mean burst probability, both because single mRNA bursts are much more frequent than coincident bursts, and because each mRNA type is probed 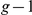 times more frequently than each pair. In practice, we therefore demand that the MLE parameters yield 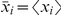 exactly.

To estimate the uncertainty in the covariances, we first note that the true variance in 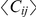 is
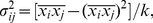
(16)where the overbar indicates the cycle or state average. We can then estimate the relevant quantity from the data, 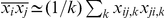, to obtain an estimate for the variance 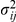. Using this estimate for 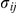, the probability of obtaining a covariance estimate 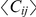 if the true covariance is 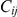 is given by:
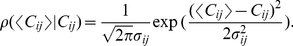
(17)Since the observations for the different mRNA pairs are independent, the likelihood of the observed covariance estimates 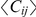 for a given set of parameters is readily obtained from Eq. (17).

### Results for cyclic dynamics

**Figure 4:**
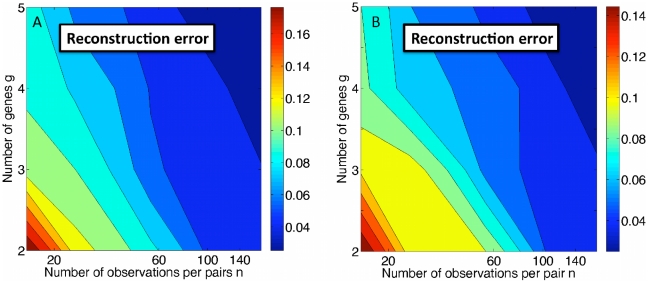
Accuracy of parameter inference for harmonically oscillating gene expression. Inference error 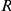 for harmonically oscillating gene expression for (A) maximum likelihood estimation (MLE) and (B) principal component analysis (PCA), in the bursty mRNA limit for different numbers of genes and FISH observations per gene pair. MLE is systematically more accurate, but only by a few percent. Averages are shown for 20 FISH data sets, generated using parameters (as defined in Eq. (6)) 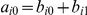, 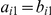, where 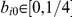, 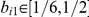 and 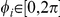 with uniform distributions. These choices ensure that all genes considered display significant variations in burst probability along the cycle and always display a positive burst probability. (On the rare occasions that a probability estimated from Eq. (6) is larger than one at some instant of time, a burst is generated in the synthetic data with a probability of one.)

As discussed above, for bursty mRNA production the means and covariances alone cannot distinguish cyclic from switching dynamics. However, if one has prior evidence that gene expression is cyclic, maximum likelihood estimation can be usefully employed to reconstruct the dynamics. If 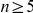 and for a sufficient data, the algorithm works well, as illustrated in Fig. 4 for harmonic 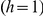 dynamics. A larger data set is needed than in the continuous mRNA regime because observations of coincident bursts are rare. Note that for 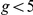, the 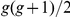 constraints from the observed means and covariances are fewer than the 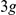 parameters, and reconstruction requires additional constraints, *e.g.* from triplet FISH data.

### Results for switching dynamics

A stochastic switch between 2 states implies a covariance matrix of rank 1, and therefore can be distinguished from cyclic dynamics, which leads to a minimum rank of 2 (unless all the genes are exactly in phase). Still, one piece of additional information is required to reconstruct the dynamics. For example, it is sufficient to know the expression level of a single gene in one state. Here, we instead assume that the probability 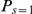 of being in one state is known, and given that constraint we infer all the levels of gene expression from synthetic FISH data. (Note that FISH data can only reveal the probabilities to be in each state, not the kinetics of switching, *e.g.* interval durations or branching ratios.) The MLE algorithm works well, as shown in Fig. 3A, as long as 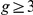 and for sufficient data. To quantify the accuracy of the MLE parameter estimation for switching dynamics, we have plotted in Fig. 3B the reconstruction error 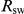 which measures the deviation of the reconstructed rates from the true rates (normalized by the state-to-state variation and weighted by the state probabilities) and averaged over all measured genes:
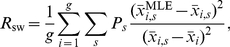
(18)where the 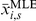 are the reconstructed rates.

In principle, with enough FISH data it should be possible to reconstruct more than just the probability of observing a burst. For example, the entire distribution of mRNAs of each type in each switching state could be obtained using MLE, *e.g.* via Expectation Maximization (EM) [26], by treating the full distributions rather than just the mean burst probabilities 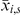 as unknowns. However, the approach proposed above of thresholding and binarizing the data has the advantage of reducing noise, and thereby reducing the required number of FISH observations, while still allowing for inference of the basic gene-expression dynamics.

### Comparison with Principal Component Analysis

In the regime of bursty mRNA production, all of the information from FISH is contained in the mean burst probabilities and the covariance matrix, suggesting that Principal Component Analysis (PCA) could be usefully applied. For example, for a 2-state switch the covariance matrix has rank 1. Thus, according to Eq. (15), performing PCA by diagonalizing 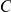 directly yields 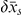, the vector of differences of burst probabilities between the two states, as the only eigenvector with a non-vanishing eigenvalue. Together with the mean burst probabilities, 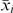, this yields full information on the switching dynamics. One caveat is that all the diagonal terms are missing from the estimated covariance matrix 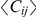, as one cannot obtain an estimate of 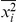 directly from FISH data. To solve this problem, we initially diagonalize the matrix 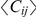 with a zero diagonal, and obtain the principal eigenvalue and eigenvector. We then approximate the diagonal terms of 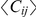 with the diagonal terms of the rank-1 matrix 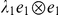 built using this single eigenvector 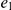. We repeat this procedure iteratively to convergence, and take the converged principal eigenvector as an estimate of 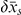. This PCA approach performs similarly well to MLE for the case of a 2-state switch, as shown in Fig. 3. The PCA approach can be easily extended to cases in which 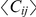 has a higher rank, where it also performs well, see Fig. 4. Of course, like MLE, PCA has the same fundamental limitations discussed above that are inherent to coincidence detection.

In practice, elements of the PCA and MLE approaches can be usefully combined. The main utility of PCA lies in diagonalizing 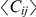 to infer its rank. (The iterative approach to filling in the diagonals of 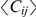 can help refine this procedure.) From the rank of 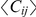, one has a direct estimate of the “complexity” of the dynamics. Complexity here means the number of states in a switch model, or the number of harmonics to be considered for cyclic dynamics. This suggests the following heuristic approach to FISH data analysis: First diagonalize 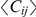. Then isolate a group of eigenvalues that are significantly larger than the rest. Use prior information to select between the different models (cyclic or switching) leading to such a rank, and finally compute the model parameters using maximum likelihood estimation.

### Applying MLE to test the putative existence of a metabolic cycle in yeast

**Figure 5:**
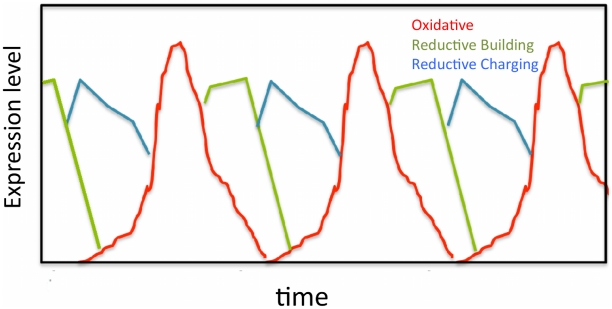
Sketch of the dynamics of the three clusters of genes: Oxidative, Reductive Building, and Reductive Charging, as identified by chemostat studies of *Saccharomyces cerevisiae* [13]. Note that the expression levels cycle periodically and approach zero at some point along the cycle. Adapted from [13].

In recent years, McKnight and coworkers demonstrated that the yeast *Saccharomyces cerevisiae* grown in chemostats can undergo synchronized metabolic oscillations [13], [27]. As shown in Fig. 5, the mRNA levels of three clusters of genes – Oxidative, Reductive Building, and Reductive Charging – were found to cycle together, with the expression of each cluster peaking at a different phase of the cycle. These population-level chemostat studies raise the question - is there an intrinsic metabolic cycle in individual cells in unsynchronized cultures? To address this question, in [14] FISH data were obtained from single, unsynchronized yeast cells. Specifically, correlations of mRNA levels were determined for pairs of genes, each of which cycled in the chemostat. The correlations observed in single cells closely matched those found in the chemostat studies, leading to the conclusion that metabolic oscillations do occur in individual cells in unsynchronized populations as well as in synchronized chemostats. However, in [14] no attempt was made to go beyond correlations to reconstruct the dynamics. Here we use MLE to infer metabolic gene dynamics in unsynchronized populations. Our results support the conclusion of [14] that the gene clusters observed in the chemostat persist in individual cells in unsynchronized cultures. In particular, we find that the genes of the Oxidative cluster oscillate together 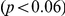 and so do the genes of the Reductive Building cluster 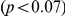. The situation is still unclear for the Reductive Charging genes, but is likely to be clarified by additional FISH data.

To analyze the dynamics, we first binarized the FISH data of [14] as appropriate for bursty gene expression. The data consists of 79 pairwise FISH experiments involving a total of 25 genes. To set an appropriate binary threshold of expression for each gene, we found the median 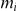 of the mRNA distribution for each gene 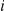. Only 7 genes have a median larger than zero (and in all cases 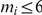), indicating that most genes are indeed bursty – despite the fact that those 25 genes were selected, among other criteria, to have a high expression level [14]. 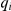 denotes the probability that the number of observed mRNA of gene 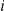 is strictly larger than 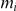 and is directly measurable from the data. We found 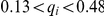, with the lower range coming from genes for which the median 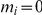.

We assumed that the dynamics is cyclic and considered the expansion of Eq. 6 up to the first harmonic. Such a model has 74 independent parameters for 25 genes. Moreover, the number of data points per pair of genes varies from 175 to 16032, with only 29 pairs having more than 2000 data points. Thus some of the correlations are well-characterized, but others are not. If only the 29 gene pairs with more than 2000 data points are considered, even a single-harmonic model is under-constrained. To circumvent this problem, we are guided by the observation apparent from Fig. 5 that the gene expression in all clusters becomes much smaller than its mean at some point in time. This suggests a simplified model where the probability 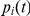 of expressing more mRNAs than the median mRNA number for gene 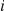 cycles as:
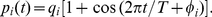
(19)Therefore, once 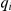 is extracted from the data there is a single free parameter per gene, namely its phase 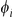.

**Figure 6:**
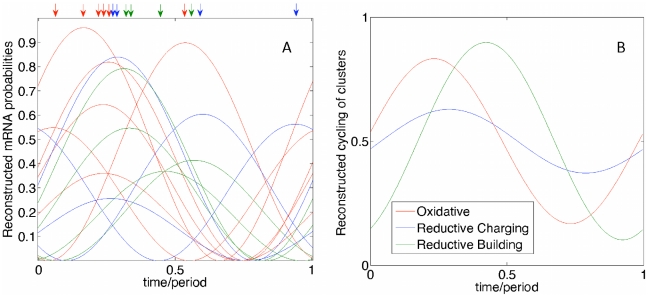
Reconstruction of the metabolic cycle. (A) Reconstructed dynamics of the probabilities 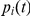 of expressing more mRNAs than the median value 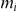. Likelihood maximization was performed on all 25 genes using all FISH data [14], but only the 14 genes with the largest number of observations are shown. There are 6 Oxidative genes (CTP1, NOP1, SNU13, SUR4, UTR2, YEF3), 4 Reductive Building genes (GAS1, HXK2, POL30, SCW10), and 4 Reductive Charging genes (CTS1, OM45, PFK26, YGP1). Genes belonging to the same clusters in Fig. 5 are represented by the same color. For each gene the position of the maximum is indicated by an arrow. (B) Average cluster activities 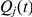 as defined in the text.

Next, the likelihood of all the observed FISH correlations was maximized with respect to the phases 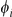. The global maximum was found by considering various random initial phases, relaxing to a maximum, repeating, and choosing the maximum with the largest likelihood. We consistently found the same maximum after the order of 10 optimization runs. Results for the reconstructed dynamics are shown in Fig. 6 for the 14 most tested genes (per gene number of observations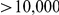). Genes belonging to each metabolic cluster identified by the chemostat studies are represented by distinct colors as indicated in the legend. The location of the maximum probability for each gene is indicated by an arrow. From the positions of the arrows it is apparent that genes belonging to the Oxidative cluster also cluster in an unsynchronized population, and so do the genes of the Reductive Building cluster. From the existing data we cannot yet conclude whether the Reductive Charging genes also cluster.

To quantify our results statistically, we define for each cluster, 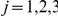, the quantity 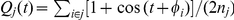, where 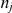 is the number of genes in cluster 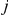. The 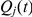, which characterize the average cluster activity, are plotted in Fig. 6. If the genes belonging to a cluster are perfectly synchronized, *i.e.* 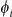 are identical for all 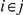, then 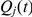 will reach zero along the cycle. More generally, the lower the minimum of 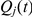, the more synchronized the cluster is for fixed 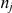. We find that the Oxidative and Reductive Building genes are indeed clustered: the probability of finding such low minima for the two corresponding curves would be only 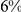 and 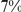 respectively (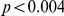 when considered together) if the phases were random. On the other hand, the minimum of the Reductive Charging cluster is comparable to the typical value for random phases.

From the chemostat studies [13], we expect the amplitudes of oscillation of metabolically cycling genes to be large (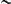
10 fold), and so global transcriptional noise (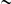
2 fold [25]) should not significantly affect our results. However, to test that our reconstruction of the metabolic cycle is robust with respect to global transcriptional noise, we reconstructed the dynamics allowing for a global correlation among mRNA levels as in Eq. (10). Specifically, we extended the model of Eq. (19) by adding the possibility of a varying global level of transcription 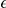:
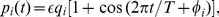
(20)where 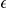 is a random variable of mean unity and standard deviation 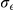. The results of the reconstruction are essentially identical to those shown in Fig. 6, where the global level of transcription was assumed to be fixed (for a comparison see Fig. S1). Moreover, from the reconstruction we infer the amplitude of the global noise of transcription to be 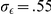 (*i.e.* 55%), which is significant, but considerably smaller than the typical variation during a cycle [13].

**Figure 7:**
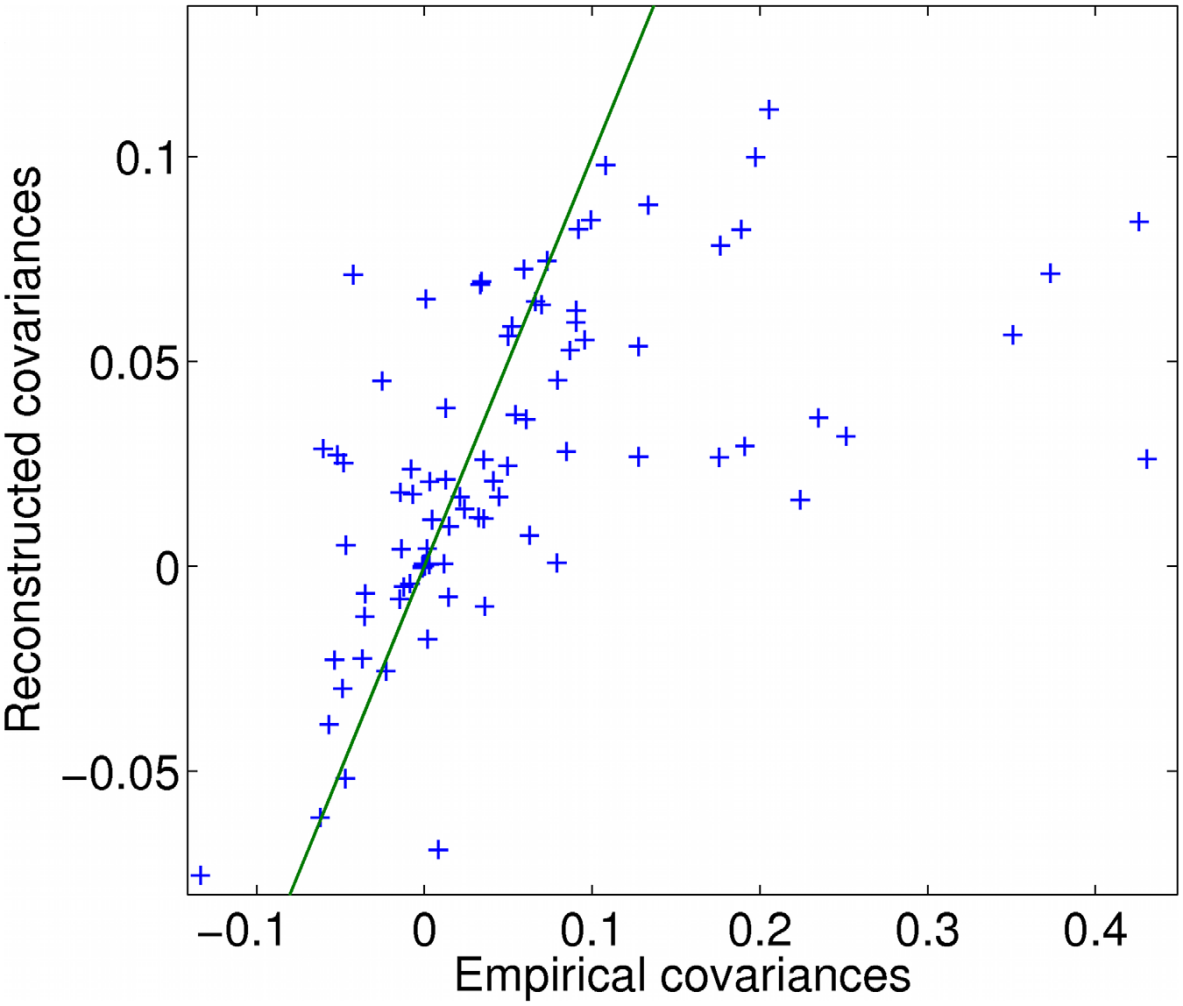
Reconstructed covariances versus observed FISH covariances for all 79 experiments in [14].

In Fig. 6, the lack of evidence for coherent oscillations of the Reductive Charging genes may reflect a real feature of unsynchronized populations. Alternatively, it may reflect the limited data and/or the simplicity of the model of Eq. (19). To investigate the limitations of this model we considered how the pairwise gene covariances it predicts compare with the observed FISH covariances, as shown in Fig. 7. Even the underlying “true” model should not capture the FISH correlations perfectly, especially since some observations are very noisy due to the limited data. However, some general trends appear. In particular our model in Eq. (19) systematically underestimates the largest covariances. This may be due to the fact that the single cosine wave that we use to fit the dynamics is less peaked than the typical expression profile observed in the chemostat [13]. Accordingly higher harmonics should be included to obtain a more accurate description of the gene-expression dynamics, an approach that will be achievable once the data set is enlarged to include additional gene pairs.

## Methods

### Finding the global likelihood maximum

In general, Maximum Likelihood Estimation (MLE) requires finding the set of model parameters for which the observed data are most likely. Finding the global maximum in the space of model parameters can be a challenging task, particularly as there may be many local maxima in which a search algorithm can get stuck. For synthetic FISH data in the regime of continuous mRNA production, we found that such local maxima occurred frequently. (In contrast, for synthetic FISH data in the bursty regime a simple steepest-descent algorithm invariably found the same maximum, independent of initial conditions.) To find the global maximum in the continuous regime, we developed a heuristic algorithm that worked very well in practice to reconstruct simple cycles.

One approach is to consider various initial parameter values, and to use a steepest-descent algorithm to find the local maximum of the likelihood. Then the global maximum (with the highest likelihood) could be chosen among the different solutions. However, in practice this procedure can be very time-consuming if initial conditions are chosen randomly. Here we propose two approaches to first compute estimates of the parameters, and then use these estimates to initiate the optimization protocol. In these two approaches we estimated the parameters as follows: (1) For the mean expression level we took 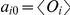. (2) For both the amplitude of oscillations 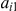 and the noise amplitude 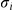 we took half the standard deviation of the observations of the corresponding gene 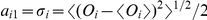. (3) Empirically we found that the initial choice of phase 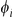 is critical in determining if the global or only a local maximum is found. Therefore, to accurately estimate the relative phases we introduced the Pearson correlation matrix (a normalized variant of our covariance matrix) 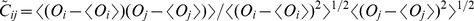. This definition implies 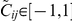. 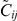 yields a rough approximation of 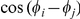, which leads to the following two approximations, the first being extremely crude:

- We assign 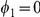. Then the vector 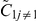 is considered. The maximum value of this vector occurs for some gene 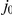, and we assign 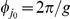. For the second maximum, at gene 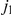, one fixes 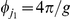, and so on. This procedure ensures that the absolute value of the relative phases between gene 1 and all other genes is approximatively correct. The main drawback is that the procedure does not prescribe the sign of the relative phases. In practice, we used this protocol twice to get two distinct initial sets of phases. To obtain the second set, we fixed 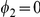, and considered the vector 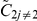.
- In the second approach, we tried to approximate the relative phases rather than their absolute values. The initial parameters 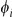 are chosen such that the matrix made of elements 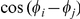 has the largest scalar product with 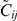, specifically, we required that 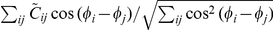 be maximized. In practice, approach (b) tended to fare better than (a).

**Figure 8:**
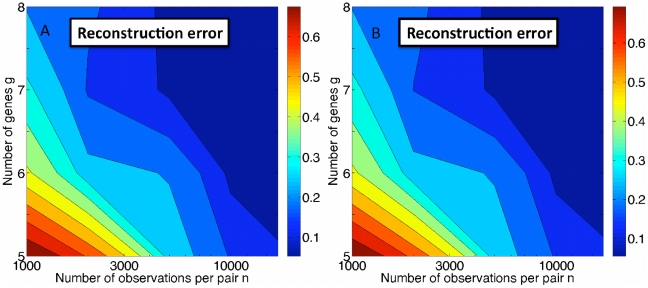
Quantification of the algorithm used to find the global maximum of the likelihood. Mean reconstruction error 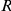 averaged over 20 realizations as the number genes 
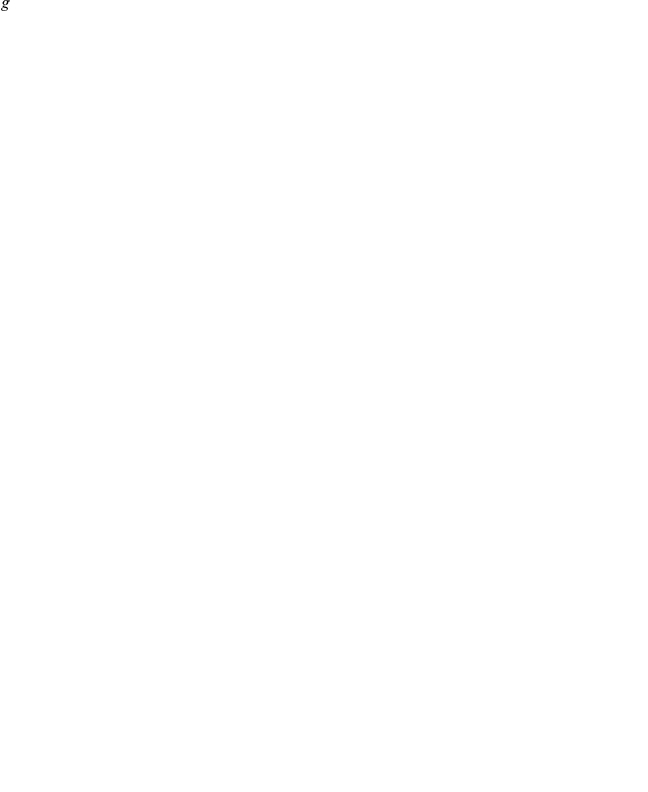
 and the number of FISH observations per gene pair 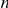 are varied for (A) the protocol described in the text and (B) for the global maximum, as shown in Fig. 2B, found by taking the parameters describing the true dynamics as initial parameters for the maximization. Results in (A) and (B) are very similar, indicating that the protocol described above generally finds the global maximum.

Results of optimization using approaches (a) and (b) to set initial parameter values are shown in Fig. 8A. The results in Fig. 8A are nearly indistinguishable from those obtained using the true parameters as initial conditions, shown in Fig. 8B, demonstrating that the above protocols performs well in identifying the global maximum of the likelihood.

## Discussion

The ability to count mRNA molecules in single cells by Fluorescence In Situ Hybridization (FISH) [9]–[11] allows for highly quantitative studies of cell-to-cell variation in gene expression. However, the requirement that cells be fixed before RNA FISH analysis precludes the use of RNA FISH to directly study transcriptional dynamics in single cells. Nevertheless, we have shown here how and when correlations between levels of different mRNAs can be exploited to reconstruct transcriptional dynamics, even if cells are asynchronous. All that is necessary is for FISH data to be obtained simultaneously for pairs of genes (or in some cases triplets of genes) a technique that is already well established [10], [12]. As a practical demonstration, we applied our approach to a large, pairwise FISH data set obtained from a recent study of the yeast *Saccharomyces cerevisiae* [14]. Our results help confirm the existence of cell-autonomous metabolic cycles in unsynchronized yeast populations [13].

To reconstruct the dynamics of gene expression from FISH data, our approach employs Maximum Likelihood Estimation (MLE) [20] to obtain the set of transcriptional parameters most likely to account for the observed data. In the regime of continuous mRNA production, apart from rescaling and inversion of time for cyclic dynamics, there is no intrinsic limit on the accuracy with which transcriptional dynamics can be reconstructed given enough data. In practice, we have shown that MLE applied to simple parameterizations for transcription (such as the leading harmonics for cyclic dynamics) allows faithful reconstruction from a moderate number of FISH observations, including noise. On the other hand, the regime where mRNA is produced in shortlived bursts [10] presents additional challenges. In this bursty regime, FISH can at most report coincidences of bursts of different mRNAs, and there are consequently fundamental limits to reconstructing the underlying dynamics. For this bursty regime, successful reconstruction will generally rely on prior knowledge regarding the class of dynamics, *e.g.* cycle vs. switch, and, even so, will in some cases require additional inputs, such as triplet FISH data. (In Table S1, we explicitly quantify the amount of such additional information required for complete dynamical reconstruction.)

In applying our approach, how should one choose among models to reconstruct gene dynamics? For example, when is it better to use multiple harmonics instead of a single harmonic to model a cycle? The answer depends on the type of data. We discuss first the regime of continuous mRNA production. For this case, a standard and reliable way to choose among models when fitting data is “leave-one-out” validation, which both rewards a good fit while punishing overfitting. In the leave-one-out approach, a model is selected and its parameters are optimized on the entire data set, but with one data point left out. The resulting parameterized model is then used to fit the neglected data point. The average fitting error, taken over all possible left-out data points, is a robust measure of the quality of the model. Among competing models, the one that minimizes this error can be selected as the better choice. In the regime of continuous mRNA production, leave-one-out validation can be applied within the MLE framework by using the log(likelihood) of the left-out data point in place of the fitting error. Among competing models, the one with the largest average log(likelihood) is the best choice.

In contrast, finding the “best” model for data in the bursty mRNA regime is generally an under-constrained problem. We showed explicitly that for many cases it is impossible in principle to distinguish among different types of models, or even to find a unique best set of parameters for a given model. Intuitively, reduction of bursty FISH data to pairwise covariances means that even as the number of FISH data points approaches infinity, the number of model constraints stays finite. So, for bursty FISH data inference alone cannot guide one in choosing the model, and one must also use common sense. Clearly, prior knowledge of the system under study should be used in selecting a model. In addition, a simple rule is that one should use models that are sufficiently parsimonious in parameters not to have degenerate solutions. For example, in analyzing FISH data on metabolic cycles, we chose the one-harmonic model because there were not enough low-noise covariances to constrain a two-harmonic model. More generally, it is advisable to choose a model with significantly more well-constrained data than parameters. If the model is barely constrained, the peak of likelihood will generally be close to flat in some directions in parameter space and the reconstruction will be poor. Figure 4 illustrates this point: 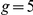 is the minimal number of genes to avoid degeneracy, but it requires 3 times more data per gene (or twice as many total data points) to reconstruct as well as for 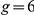. In practice, one test for the quality of the reconstruction in the bursty regime is to compare the observed covariances to the reconstructed covariances, as shown in Fig. 7 for the case of the yeast metabolic cycle genes.

Reconstruction of gene-expression dynamics from FISH data presents multiple practical challenges. One important issue is noise in the measurement of mRNA levels. For the regime of continuous mRNA production, we have shown that sufficient data can compensate for both the noise inherent in gene expression and the noise arising from uncertainty in measurement. For the regime of bursty mRNA production, “binarizing” the data into the presence or absence of a significant number of mRNA molecules substantially reduces the impact of measurement noise. A practical question here is the best threshold to use for binarizing the data. In many cases, the dynamics will be best reconstructed by setting the threshold well above 1 mRNA transcript; for example, in treating the data for metabolic cycles we chose the median expression level for each gene as its threshold. A higher threshold is less sensitive to measurement noise (fewer false positives), and to occasional transcripts produced by promoter leakage (better identification of true bursts), and a higher threshold also allows finer time resolution, as a given burst will remain above threshold for a shorter time (*e.g.* preventing blurring of boundaries between switching states). However, a higher threshold reduces the number of coinciding bursts in the data, requiring more overall FISH observations. An important related issue is the possibility of correlated noise in the transcription of different genes. An example of such noise is the observed global correlation among transcription rates in yeast [25]. Fortunately, global noise can be readily incorporated within the MLE framework by introducing a single additional variable in the model for gene expression, as in Eq. (10). Indeed, our treatment of global noise among genes involved in the yeast metabolic cycle yields an independent, and reasonable, estimate for this noise at 55% of mean expression. (More complex noise correlations among different genes would require case-by-case analysis.)

False-positive rates and false-negative rates are also both important considerations in analyzing FISH data. These are essentially technical issues beyond the scope of our study, but a few remarks are in order. In Ref. [14], both false positives and false negatives were reduced by the use of multiple fluorescent probes (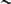
5) for each mRNA. Only high-contrast spots above a fluorescence threshold indicative of multiple bound probes were counted. This threshold was set empirically from the fluorescence distribution of spots outside of cell boundaries, corresponding to single probes. Nevertheless, with any such thresholding method, there will be cases where the “presence” or “absence” of an mRNA is ambiguous, and in the bursty regime such ambiguities can strongly impact the binarization of the data. Fortunately, because MLE is an intrinsically probabilistic approach, ambiguities can be dealt with by treating the two possibilities, present or absent, probabilistically. As in Ref. [14], by looking at spots outside of cell boundaries, one can obtain the distribution of intensities for spots that are actually noise (typically single probes that have not been washed away), and by looking inside cell boundaries a similar distribution can be obtained for spots that correspond to real mRNAs (multiple probes). Spots inside cells that fall into the region of overlap of these two distributions can then be assigned the corresponding probabilities of being present (real) or absent (noise). MLE can then incorporate both possible interpretations of the data, with their appropriate weights, in the data set.

A related issue, highlighted by Zenklusen *et al.* [11], is the existence of nascent mRNA transcripts at the locus of the gene. In the regime of continuous mRNA production, an estimate of nascent transcript number, possibly non-integer, could simply be added to mRNA counts. In the bursty regime, the existence of such transcripts might well be taken as *prima facie* evidence for active transcription, and therefore treated as equivalent to the presence of an above-threshold burst.

Another practical issue in reconstructing gene-expression dynamics from FISH measurements is that data may come in mixed forms, *e.g.* pairwise FISH data+triplet FISH data+additional constraints or prior information. Again, MLE is naturally suited to incorporating mixed data types since all sources of information can be combined to produce the overall likelihood of the data given a set of model parameters, including prior information on the model parameters themselves (*cf.* Eq. (2)).

While these and other practical issues are important to consider, our successful reconstruction of yeast metabolic cycles using the FISH data of Silverman *et al.* [14] demonstrates that our approach can provide a useful tool for analyzing gene-expression dynamics. In fact, our analysis of this data raises several new questions. First, since our reconstruction was statistically significant for the Oxidative and Reductive Building clusters but not for the Reductive Charging cluster, it is possible that cycles of the latter may be weaker in unsynchronized cultures than in synchronized chemostats. Second, our reconstruction indicates a spread among the oscillatory phases of genes within each cluster – is this spread a consequence of the limited data, or are the oscillation patterns of genes within clusters distinct? We expect that additional FISH data coupled with MLE analysis will soon provide answers to these questions.

The many advantages of FISH – absolute quantification, high time resolution, use of wild-type cells, ability to simultaneously measure multiple mRNA types, and broad application across species from bacteria [28] to yeast [11], [14] to metazoans [9], [10], suggest that FISH will find many uses in future studies of gene expression, including applications beyond those currently demonstrated. For example, FISH can be applied to cells in structured environments such as tissues or biofilms, or even cells in mixed-species consortia. In all of these cases, population level studies of gene expression cannot reveal the important cell-to-cell variations. Of course, FISH is not the only technique that yields quantitative snapshots at the single-cell level. Immunofluorescence and single-cell sequencing also meet the requirements of simultaneous measurements of two or more intracellular factors. We hope that the analysis presented here can facilitate the application of FISH and other single-cell snapshot assays to cases where both cell-to-cell variation and the dynamics of gene expression are of central interest.

## Supporting Information

Figure S1 Average cluster activities Q_j_(t)as defined in the text, taking into account the presence of global transcriptional noise.
(0.96 MB TIF)

Table S1 Rank of covariance matrix and required number of additional constraints (obtained from triplet-FISH measurements or other sources) necessary for complete parameter inference in the regime of bursty mRNA production, for both cyclic and stochastic switching dynamics.
(0.44 MB TIF)
