# Supplementary figures and images for: Evaluating Gene Expression Dynamics Using Pairwise RNA FISH Data
Source: PLoS Comput Biol. 2010 Nov 4;6(11):e1000979. doi: 10.1371/journal.pcbi.1000979 (PMC2973809; doi:10.1371/journal.pcbi.1000979)

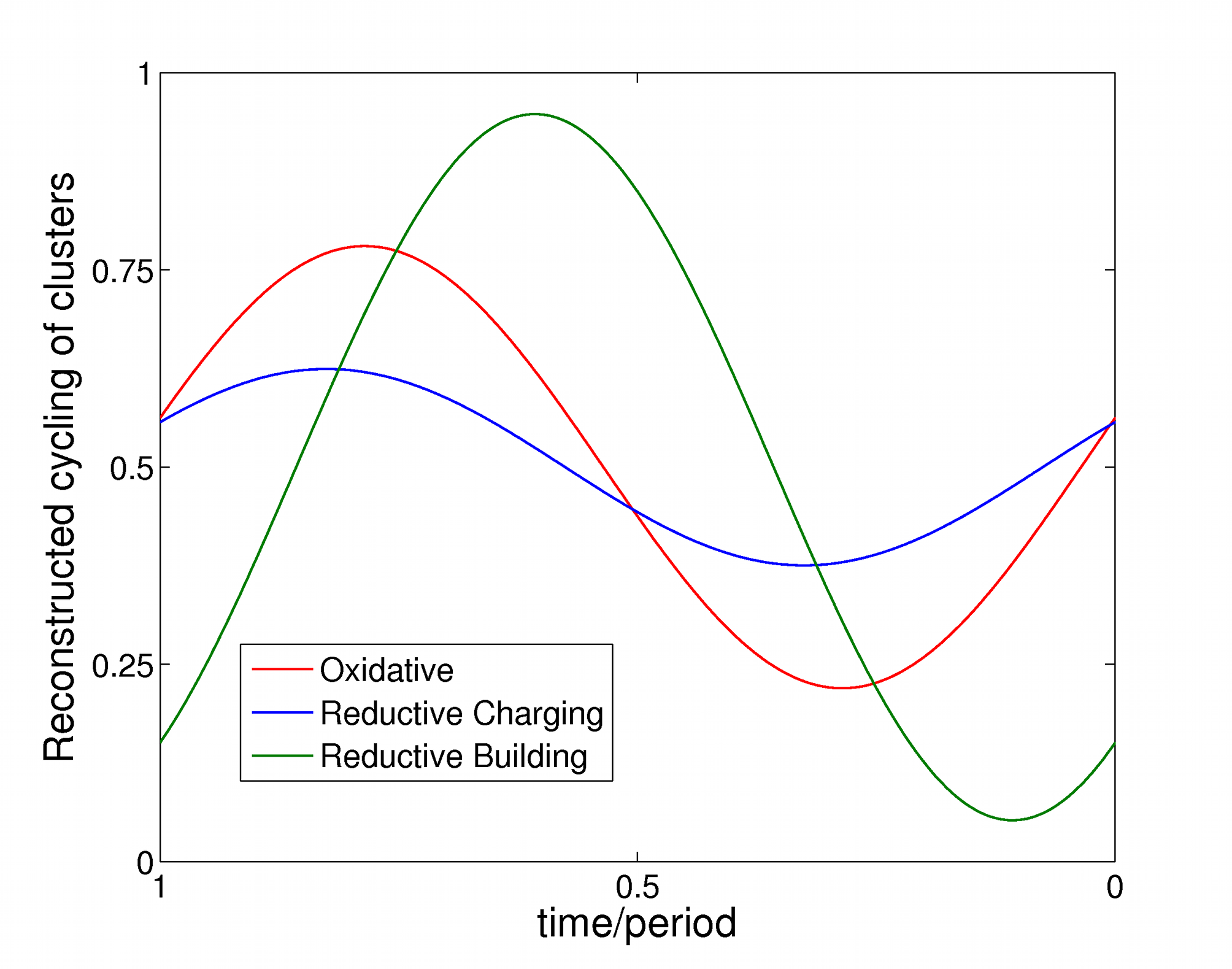

Supplement: Figure S1 — Average cluster activities Qj(t)as defined in the text, taking into account the presence of global transcriptional noise. (0.96 MB TIF) [file pcbi.1000979.s001.tif]

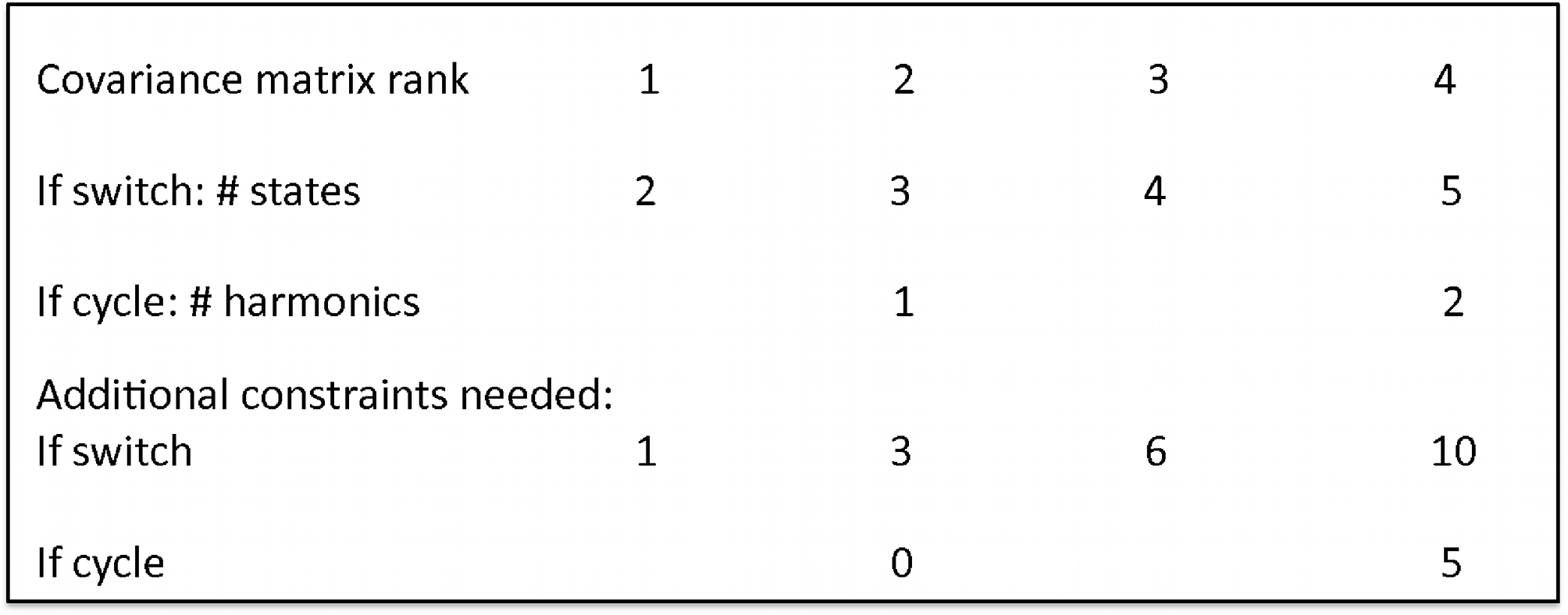

Supplement: Table S1 — Rank of covariance matrix and required number of additional constraints (obtained from triplet-FISH measurements or other sources) necessary for complete parameter inference in the regime of bursty mRNA production, for both cyclic and stochastic switching dynamics. (0.44 MB TIF) [file pcbi.1000979.s002.tif]
